# Characteristics of subretinal particles detected after pars plana vitrectomy for rhegmatogenous retinal detachment

**DOI:** 10.1186/s12886-023-02865-w

**Published:** 2023-03-23

**Authors:** Sho Noji, Masaharu Mizuno, Makoto Inoue, Takashi Koto, Akito Hirakata

**Affiliations:** Kyorin Eye Center, Kyorin University School of Medicine, 6-20-2 Shinkawa, Mitaka, Tokyo, 181-8611 Japan

**Keywords:** Fundus autofluorescence, Rhegmatogenous retinal detachment, Subretinal fluid, Vitrectomy, Subretinal particles

## Abstract

**Background:**

To determine the incidence and characteristics of the multiple subretinal particles (SRPs) present after pars plana vitrectomy (PPV) for rhegmatogenous retinal detachment (RRD).

**Methods:**

The medical records of 224 eyes of 224 patients that underwent PPV for RRD were reviewed. The presence of SRPs in the subretinal fluid blebs and the presence of subretinal deposits were determined by optical coherence tomography (OCT) and fundus autofluorescence (FAF). The characteristics of the FAF and infrared reflectance (IR) images of a scanning laser ophthalmoscope in eyes with SRPs (SRPs group) were compared to that of eyes without SRPs (control group).

**Results:**

SRPs were observed in 27 eyes (12%), and they were completely resolved in 20 eyes (74%) after 6 months. The incidence of macula-off RRD (85%) and preoperative precipitates (41%) were significantly higher in the SRPs group than that in the control group (64%, *P* = 0.046; 12%, *P* = 0.002). The axial length was significantly shorter in the SRPs group than that in the control group (25.04 ± 1.54 mm, 26.00 ± 1.78 mm, *P* = 0.012). The preoperative and postoperative best-corrected visual acuity were not significantly different between the two groups (*P* = 0.702, *P* = 0.337). The subretinal fluid bleb determined by OCT were hyperfluorescent in the FAF images in 24 eyes (89%), and the subretinal deposits were hypofluorescent with solid appearance by OCT other than fluid in 3 eyes (11%). The hypofluorescent subretinal deposits in the FAF images were bright in the IR images in 2 eyes.

**Conclusions:**

The SRPs consist of lipofuscin-related hyperfluorescent subretinal fluid and the subretinal deposits containing bright IR melanin particles of proliferating retinal pigment epithelial cells.

**Supplementary Information:**

The online version contains supplementary material available at 10.1186/s12886-023-02865-w.

## Background

Scleral buckling surgery and pars plana vitrectomy (PPV) have been used to treat eyes with rhegmatogenous retinal detachments (RRDs) [1–3]. A foveal reattachment is attained in a high percentage of the cases soon after the PPV for macular-off RRD [4]. However, subretinal fluid may develop and persist for several months in some eyes [2–10]. Some studies have reported that the persistent subfoveal fluid did not affect the final visual outcome [11, 12], however other studies have reported that it can lead to metamorphopsia and a reduction of the visual acuity [2, 3].

Persistent subretinal fluid after surgery for a RRD has been described initially as focal pockets of turbid subretinal fluid that resembled retinal pigment epithelium (RPE) detachments [13]. Fu and associates [11] divided the persistent subretinal fluid into three types; bleb-like loculated fluid, shallow-diffuse fluid, and multiple blebs from their appearance in optical coherence tomographic (OCT) images. Subretinal fluid blebs were also observed in 9.3% of the eyes after scleral buckling surgery with choroidal vascular congestion and hyperpermeability in the indocyanine green angiograms [14]. Subretinal fluid blebs were observed in 21.6% of the cases as dark round spots in the infrared reflectance (IR) scanning laser ophthalmoscopic (SLO) images after successful RRD reattachment by vitrectomy or scleral buckling surgeries [15]. The subretinal fluid blebs were also reported to be present in 7.4% of the eyes after RRD surgery and were seen as hyperfluorescent spots in the lipofuscin-related fundus autofluorescence (FAF) images and dark spots in the IR images [16].

We recently had a patient with a RRD who had greenish particles in the subretinal space intraoperatively that moved in the fluid during PPV. The particles were displaced away from the fovea by the intravitreal injection of perfluorocarbon liquid. We assumed that these subretinal particles probably included several types of tissues other than the subretinal fluid. However, the exact identification of these particles was not made.

Thus, the purpose of this study was to determine the incidence and characteristics of the subretinal particles that are detected after pars plana vitrectomy (PPV) for a RRD.

## Methods

We reviewed the medical records of patients who had undergone PPV (25- or 27-gauge) to treat a RRD in our hospital between April 2019 and August 2020. All of the patients were followed for at least 6 months. Subretinal particles (SRPs) were detected by ophthalmoscopy, optical coherence tomography, and widefield FAF. A detailed clinical history that included the date of the onset of the visual disturbances and history of other ocular diseases were obtained at the initial examination. The clinical characteristics of the patient including the age, sex, axial length, interval between the onset of the symptoms to the time of surgery, baseline status of the macula, and the best-corrected visual acuity (BCVA) before and after the surgery were obtained from the medical records. The OCT images (Heidelberg Retina Angiograph 2, Heidelberg), widefield FAF images (200Tx®, California or Silverstone, Optos Inc, Dunfermline, UK), and IR images of the confocal scanning laser ophthalmoscope (Heidelberg Retina Angiograph 2, Heidelberg) were compared between eyes with SRPs (SRPs group) and eyes without SRPs (control group). The BCVA was determined by a Landolt C chart, and the decimal BCVAs were converted to the logarithm of the minimum angle of resolution (logMAR) units for the statistical analyses. The axial length was measured by a swept-source optical biometer (OA-2000, TOMEY Corp., Nagoya, Japan).

All of the patients received a detailed explanation of the surgical and ophthalmic examination procedures, and all signed an informed consent form. The procedures adhered to the tenets of the Declaration of Helsinki, and they were approved by the Institutional Review Committee of the Kyorin University School of Medicine. All of the patients consented to our review and anonymized publication of their findings.

### Surgical procedures

All patients underwent 25- or 27-gauge PPV (Constellation® Vision System; Alcon Laboratories Inc, Fort Worth, TX) using a widefield viewing system (Resight®; Carl Zeiss Meditec, Oberkochen, Germany) under topical and sub-Tenon anesthesia. Combined PPV and phacoemulsification with implantation of an intraocular lens were performed on patients who were ≥ 50-years-of-age or at the discretion of the surgeon. Forty milligrams of triamcinolone acetonide (TA; MaQaid®; Wakamoto Pharmaceutical Co., Ltd., Tokyo, Japan) was mixed with 2.0 ml of balanced salt solution to produce a TA suspension. The TA suspension was injected intravitreally to make the vitreous cortex more visible, and a posterior vitreous detachment (PVD) was created as completely and as far peripherally as possible if it was not present. The peripheral vitreous was shaved with scleral indentation. The subretinal fluid was drained through pre-existing retinal breaks after fluid/air exchange. Perfluorocarbon liquid was used at the surgeons’ discretion. Laser photocoagulation was then performed around the retinal breaks. Scleroconjunctival sutures were applied with 7–0 vicryl (polyglactin 910) when leakage from the sclerotomies was detected. Then, 10% or 20% sulfur hexafluoride (SF_6_) was injected as a gas tamponade. Patients were instructed to maintain a face-down or lateral posture to tamponade the retina.

### Statistical analyses

The significance of the differences in the baseline characteristics, surgical procedures, and surgical outcomes between the 2 groups was determined by Mann–Whitney *U* tests or Fisher’s exact possibility tests. Multivariate logistic regression analyses were used to eliminate the effects of the confounding factors to determine the baseline characteristics. All analyses were performed using the R statistical software (version 4.0.5, The R Foundation for Statistical Computing, Vienna, Austria). A *P* of < 0.05 was taken to be statistically significant.

## Results

**Fig. 1:**
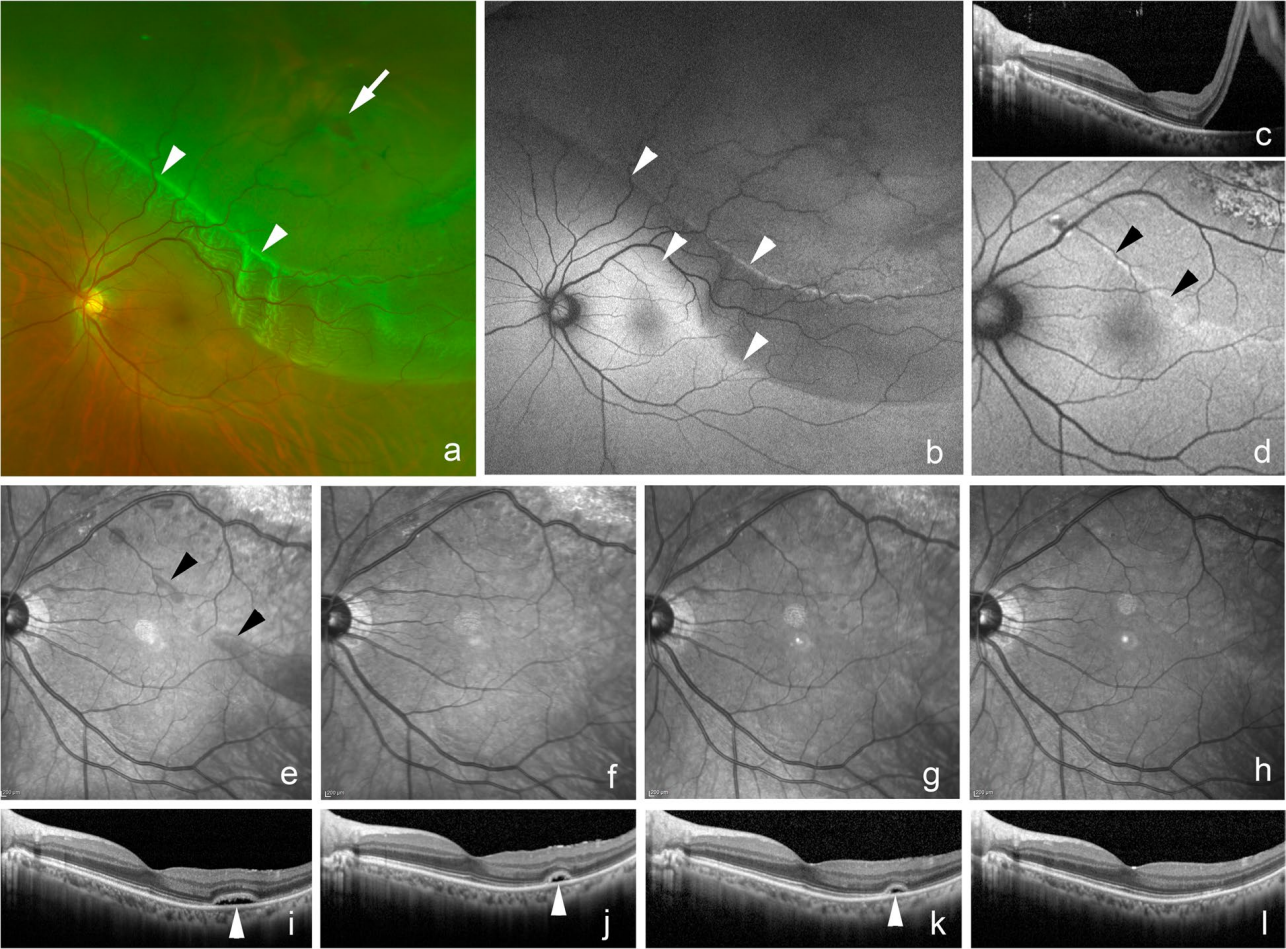
Fundus photographs and optical coherence tomographic (OCT) images of a 51-year-old man with subretinal fluid blebs after vitrectomy for a rhegmatogenous retinal detachment (RRD) and a vision of 20/20. **a**: Preoperative widefield image showing a superior RRD with a retinal break (arrow) and a subretinal band (arrowheads). **b**: Preoperative fundus autofluorescence (FAF) image shows a hypofluorescent area corresponding the area of the RRD and hyperfluorescent area (arrowheads) at the subretinal band and the inferior edge of the RRD **c**: Preoperative OCT image of a horizontal scan showing that the RRD extends to the macula. **d**: Postoperative FAF image at 1 month showing a hyperfluorescent band (arrowheads) at the superior side of the macula and absence of a hypofluorescent area corresponding to the preoperative RRD. **e**: Postoperative infrared (IR) image at 1 month showing a dark band (arrowheads) on the temporal side of the macula corresponding to the hyperfluorescent area in FAF image (**d**) and persistent subretinal fluid (arrowhead) in the horizontal OCT (**i**). **f**: Postoperative IR image at 3 months showing an absence of the dark band area and at 6 months (**g**) and 12 months (**h**). **j**: Postoperative horizontal OCT at 3 months and 6 months (**k**) showing the persistent subretinal fluid (arrowhead) and its disappearance at 12 months (**l**)

A total of 224 eyes of 224 patients (155 men and 69 women; mean age of 59.1 ± 9.5 years) were studied. The retina was reattached after the initial surgery in 220 eyes (98%) and finally in all eyes. SRPs were observed in 27 eyes (12%, Table 1, Fig. 1). The BCVA was significantly improved after 6 months in the SRPs group (0.48 ± 0.44 logMAR units to 0.04 ± 0.12 logMAR units, *P* < 0.0001), and also in the control group (0.64 ± 0.69 logMAR units to 0.06 ± 0.22 logMAR units, *P* < 0.0001). The pre- and postoperative BCVA at 1 and 6 months were not significantly different between the two groups (preoperative; *P* = 0.702, postoperative 1 month; *P* = 0.548, 6 months; *P* = 0.337, respectively).

**Table 1:** Characteristics of the subjects with or without multiple subretinal particles (MSPs)

	MSPs	control	*p* value
Cases (eye) (%)	27 (12%)	197 (88%)	-
Age (year)	60.0 ± 8.9	58.9 ± 9.5	0.745^†^
Gender (man/woman)	18 / 9	137 / 60	0.825^‡^
Macular off detachment	23 (85%)	127 (64%)	0.046^‡^
Duration of symptoms (day)	14.6 ± 15.3	11.2 ± 11.6	0.252^†^
Axial length (mm)	25.04 ± 1.64	26.00 ± 1.78	0.012^†^
Subretinal precipitate	11 (41%)	24 (12%)	0.002^‡^
PPV (25G/27G)	25 / 2	194 / 3	0.105^‡^
Combined cataract surgery	25 (93%)	164 (83%)	0.089^‡^
Intentional retinal break	1 (4%)	3 (2%)	0.393^‡^
Preop BCVA (logMAR)	0.48 ± 0.44	0.64 ± 0.69	0.702^†^
Postop BCVA (1 month)	0.17 ± 0.22	0.18 ± 0.31	0.548^†^
Postop BCVA (6 months)	0.04 ± 0.12	0.06 ± 0.22	0.337^†^

*MSPs* Multiple subretinal particles, *PPV* Pars plana vitrectomy, *BCVA* Best-corrected visual acuity, *logMAR* Logarithm of the minimum angle of resolution

^†^Mann–Whitney *U* test, ^‡^Fisher’s exact probability test

The size of the area where the SRPs were located decreased over time, and the SRPs were completely absent in 20 eyes (74%) at 6 months postoperatively (Fig. 1). The incidence of macula-off RRD (85%) and presence of preoperative precipitates (41%) were significantly higher in the SRPs group than in the control group (64%, *P* = 0.046 vs 12%, *P* = 0.002, respectively). The axial length was significantly shorter in the SRPs group than in the control group (25.04 ± 1.54 mm vs 26.00 ± 1.78 mm, *P* = 0.012, respectively). The age, sex distribution, duration of the symptoms, gauge of instruments for vitrectomy, incidence of combined cataract surgery, and cases with intentional retinal breaks were not significantly different between the two groups.

**Fig. 2:**
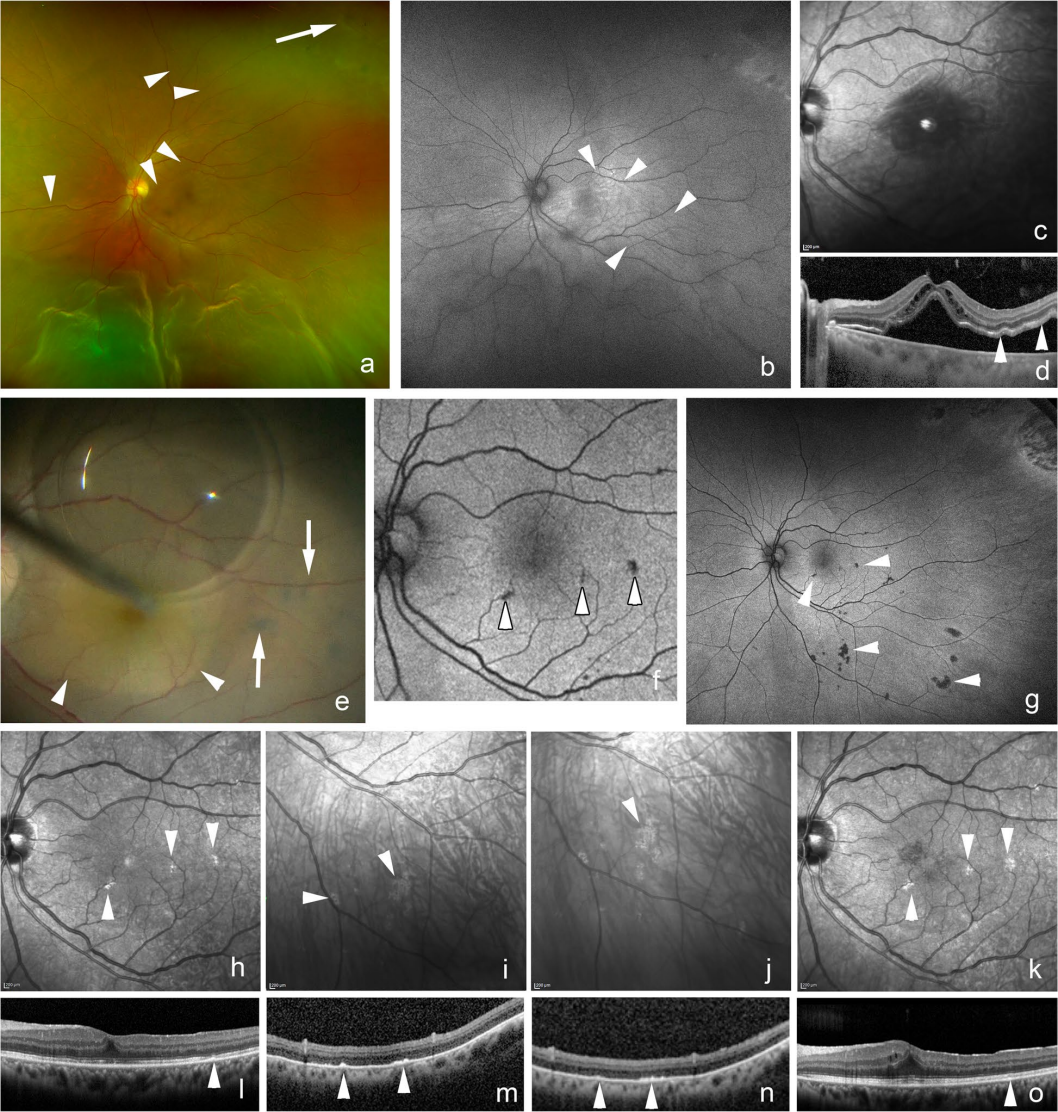
Fundus photograph and OCT images of a 53-year-old man with subretinal deposits after vitrectomy for a rhegmatogenous retinal detachment (RRD). **a**: Preoperative widefield image showing superior and inferior bullous RRD (arrowheads) with a superior retinal break (arrow). Vision is 20/50 due to the macula-off RRD. **b**: Preoperative fundus autofluorescence (FAF) image showing a hypofluorescent area corresponding the area of RRD and hyperfluorescent wrinkles (arrowheads) at the posterior pole and the inferior edge of the RRD. **c**: Preoperative infrared (IR) image showing a dark area corresponding to the RDD at the macula and temporal and inferior quadrants. **d**: Preoperative optical coherence tomographic image of a horizontal section showing a macula-off RRD with elongations of the outer segments (arrowheads) of the detached retina. **e**: Intraoperative image after internal limiting membrane peeling (arrowheads) with assist of Brilliant Blue G showing green deposits (arrows) in the subretinal space and perfluorocarbon liquid helped to move the subretinal deposits away from the macula (Supplemental videoclip-1). **f**: Postoperative FAF image at 1 month showing hypofluorescent spots (arrowheads) inferior to the macula after retinal reattachment. **g**: Postoperative FAF image at 6 months showing persistent hypofluorescent spots (arrowheads) inferior to the macula and in the inferior quadrants. **h**: Postoperative IR image at 1 month showing bright spots (arrowheads) corresponding to the hypofluorescent spots in the FAF image (**f**) and hyperreflective subretinal deposits (arrowheads) in the horizontal OCT (**l**) with irregular ellipsoid zone band in this area. **i**: Postoperative IR images at 3 months and 6 months (**j**) showing bright spots (arrowheads) corresponding to the hyperreflective subretinal deposits (arrowheads) in the horizontal OCT images (**m** and **n**). **k**: Postoperative IR image at 12 months showing persistent bright spots (arrowheads). Vision has improved to 20/25. o: Postoperative horizontal OCT image at 12 months showing reduced hyperreflective subretinal deposits (arrowheads) with improvement of ellipsoid zone band in this area

**Fig. 3:**
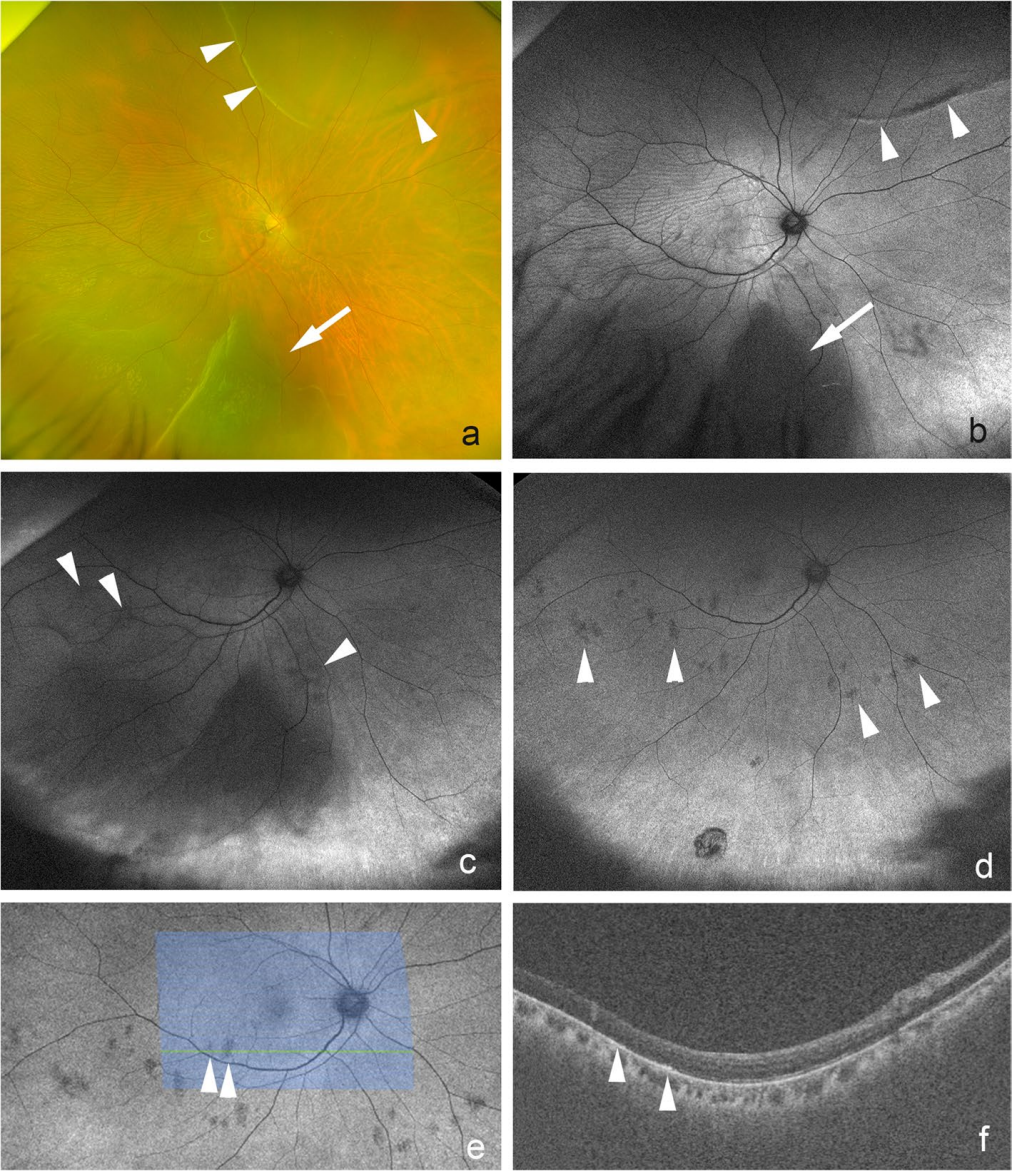
Fundus photographs and optical coherence tomographic images of a 54-year-old man with subretinal deposits after vitrectomy for a rhegmatogenous retinal detachment (RRD). **a**: Preoperative widefield image showing a superior and inferior bullous RRD with a superior retinal break within a demarcation line (arrow). Vision has decreased to 20/50 due to the macula-off RRD. **b**: Preoperative fundus autofluorescence (FAF) image showing hypofluorescent area corresponding to the RRD (arrow) and hyperfluorescent area (arrowheads) at the demarcation line and the inferior edge of the RRD. **c**: Preoperative fundus FAF image showing hypofluorescent particles (arrowheads) within the area of retinal detachment **d**: Postoperative FAF image at 4 months showing multiple hypofluorescent particles (arrowheads). **f**: Postoperative horizontal OCT image at 2 months showing hyperreflective subretinal deposits (arrowheads) corresponding to the hypofluorescent particles (e, arrowheads) in monitor FAF image of Silverstone OCT

The SRPs were hyperfluorescent in the FAF images in 24 eyes (89%, Fig. 1) and hypofluorescent in 3 eyes (11%, Figs. 2 and 3). The hyperfluorescent areas were observed to correspond to the persistent subretinal fluid in the OCT images and were dark in the IR images. The hypofluorescent areas in the FAF images were observed as solid subretinal deposits in the OCT images and the hypofluorescent areas of the 2 eyes were bright in the IR images (*P* = 0.003, Table 2). The age, sex, incidence of macula-off RRD, duration of symptoms, axial length, presence of subretinal precipitates and subretinal band, gauge of the surgical instruments for the vitrectomy, incidence of combined cataract surgery, intentional retinal break, preoperative and postoperative BCVA were not significantly different between the two groups.

**Table 2:** The fundus autofluorescence (FAF) findings in eyes with multiple subretinal particles

FAF	Hypo	Hyper	*P* Value
Eyes	3 (11%)	24 (89%)	-
IR (bright/dark)	2 / 0	0 / 24	0.003^‡^
Age (years)	54.0 ± 0.8	60.7 ± 9.2	0.289^†^
Gender (man/woman)	3 / 0	15 / 9	0.529^‡^
Macular off detachment	2 (67%)	4 (17%)	1^‡^
Duration of symptoms (day)	23.3 ± 26.0	13.5 ± 13.0	0.884^†^
Axial length (mm)	25.44 ± 2.22	24.99 ± 1.54	0.812^†^
Subretinal precipitate	2 (67%)	9 (38%)	1^‡^
Subretinal bands or demarcation lines	1 (33%)	6 (25%)	1^‡^
PPV (25G/27G)	3 / 0	22 /2	1^‡^
Combined cataract surgery	1 (33%)	24 (100%)	0.077^‡^
Intentional retinal break	0 (0%)	1 (4%)	1^‡^
Preop BCVA (logMAR)	0.32 ± 0.20	0.50 ± 0.46	0.530^†^
Postop BCVA (1 month)	0.06 ± 0.10	0.18 ± 0.23	0.265^†^
Postop BCVA (6 months)	-0.01 ± 0.05	0.05 ± 0.12	0.518^†^

*FAF* Fundus autofluorescence, *hypo* Hypofluorescent, *hyper* Hyperfluorescent, *IR* Infrared reflectance, *BCVA* Best-corrected visual acuity, *logMAR* Logarithm of the minimum angle of resolution

^†^Mann–Whitney *U* test, ^‡^Fisher’s exact probability test

Multivariable regression analyses showed that the incidence of SRPs was significantly higher in eyes with macula-off RRD (*P* = 0.006, Table 3). In addition, SRPs were present significantly more frequently in eyes with preoperative precipitates (*P* = 0.002) and shorter axial lengths (*P* = 0.037) than in eyes of the control group.

**Table 3:** Multivariable logistic regression analysis in eyes with or without multiple subretinal particles (MSPs)

	MSPs	control	Odds ratio	95% CI	*P* value
Cases (eye) (%)	27 (12%)	197 (88%)	-	-	-
Age (year)	60.0 ± 8.9	58.9 ± 9.5			0.442
Gender (man/woman)	18 / 9	136 / 61			0.724
Macular off detachment	23 (85%)	127 (64%)	0.163	0.045—0.593	0.006
Duration of symptoms (day)	14.6 ± 15.3	11.2 ± 11.6			0.587
Axial length (mm)	25.04 ± 1.64	26.00 ± 1.78	1.4	1.02—1.93	0.037
Subretinal precipitate	11 (41%)	24 (12%)	4.75	1.79—12.6	0.002
Preop BCVA (logMAR)	0.48 ± 0.44	0.64 ± 0.69			0.096
Postop BCVA (1 month)	0.17 ± 0.22	0.18 ± 0.31			0.863
Postop BCVA (6 months)	0.04 ± 0.12	0.06 ± 0.22			0.722

*MSPs* Multiple subretinal particles, *BCVA* Best-corrected visual acuity, *logMAR* Logarithm of the minimum angle of resolution

## Discussion

The incidence of multiple subretinal fluid blebs varied with the surgical procedures performed. Kang and associates [14] reported that the incidence of multiple subretinal fluid blebs was 9.3% after scleral buckling and cryopexy. They also reported that indocyanine green angiography demonstrated choroidal vascular congestion and hyperpermeability near the subretinal fluid blebs. Otsuka and associates [16] reported that the incidence of multiple subretinal fluid blebs was 7.4% at one month after 27-gauge PPV. However, the incidence of multiple subretinal fluid blebs differed by the type of retinal detachment including macula-on or macula-off detachments, duration of the retinal detachment, external drainage with scleral buckling, internal drainage, or drainage from the retinal breaks during vitrectomy.

Our results showed that the incidence of the SRPs was 12%, and they were detected after either 25-gauge or 27-gauge vitrectomy. The incidence of SRPs was higher in eyes with macular-off RRD, presence of preoperative subretinal precipitates, and shorter axial lengths. The subretinal fluid blebs among the SRPs appeared to be persistent viscous subretinal fluid which was present because of the long-standing RRD. Less liquified vitreous fluid due to the shorter axial length of the eye also contributed to the persistent subretinal fluid. Fu and associates [11] described the shallow and diffuse pattern of the persistent subretinal fluid after scleral buckling surgery for RRD, and they transformed to the multiple blebs pattern in 69% of the eyes at 12 months after the surgery. Then, the size and numbers of blebs decreased gradually until all were completely absorbed. An increase in the optical reflectivity of the OCT images in eyes with persistent subretinal fluid after surgical repair of the macula-off retinal detachment has been reported [17]. An increase in the glutamate level and cell-rich components of the subretinal fluid have been reported in eyes with persistent subretinal fluid [18, 19]. The SRPs were present in 24 eyes with multiple subretinal fluid blebs and 3 eyes with subretinal deposits were detected in the OCT images. The SRPs had characteristics of multiple subretinal fluid blebs because they mainly consist of multiple subretinal fluid blebs.

Kim and associates [15] reported that subretinal fluid blebs were detected more frequently in younger patients, in phakic eyes, in eyes with macula-off RRD (87.5%), and after scleral buckling surgery (70%) than after PPV (30%). The height of the subretinal blebs was greatest at 2.9 postoperative months and the width of the subretinal fluid blebs gradually decreased over time. These changes indicated that the subretinal fluid blebs originated mainly from the activity of the reattachment of the RPE layer to the photoreceptors during the resolution of the RRD [15]. A larger width-to-height ratio of the subretinal fluid bleb indicated a higher risk of persistent subretinal fluid beyond 6 months [20].

Subretinal precipitates have been observed as cream-colored aggregates of cell clumps in the subretinal space in eyes with retinal detachment [7]. Analysis of the subretinal fluid from cases of RRD showed a positive correlation between the protein concentration and the duration of the detachment but was not correlated with the presence of subretinal precipitates [7]. Electron microscopy has identified the cellular structures in the subretinal space as pigment-laden macrophages that originated from the RPE cells [7]. Our results also indicated a strong correlation between the presence of subretinal precipitates and eyes with SRPs. Veckeneer and associates [21] studied subretinal fluid samples collected during RD repair surgery and found photoreceptors, photoreceptor fragments, RPE cells, and red blood cells in the samples. The persistent subretinal fluid with elongated photoreceptor outer segments has been suggested to be the cause of the hyperfluorescence of the FAF images [22, 23].

The hypofluorescent lesions in the FAF images in 3 eyes in the SRPs group appeared to be solid subretinal deposits in the OCT images, and the lesions were bright in the IR images in 2 eyes. The subretinal proliferation of the RPE cells with melanin granules may cause the hyperreflective IR areas. Ly and associates [24] described the hyperreflective areas of the IR images as small and medium-sized drusen and pigmentary changes and scattered hyperreflectivity in occult choroidal neovascularization in eyes with age-related macular degeneration. Venkatesh and associates [25] reported hyperreflectance of the IR images in eyes with choroidal nevi. Banda and associates [25] reported on the efficacy of widefield infrared reflectance imaging in eyes with retinoschisis, retinal detachment, and retinal schisis detachments [26]. The edge of the retinal detachment and edge of the retinal schisis detachment with RPE changes appeared to be hyperreflective in the IR images [26]. The reactive proliferation of RPE cells most likely led to the IR-reflectivity which may offset the fluid-associated absorption. Weinberger and associates [27] reported that the near-infrared autofluorescent images observed in age-related macular degeneration and central serous choroidopathy were highly correlated with the similar near-infrared reflectivity as near-infrared autofluorescence contributed to the near-infrared reflectivity derived from melanin. The SRPs with hypofluorescent FAF were correlated to the subretinal proliferation of RPE cells with melanin-related bright IR reflectivity. We assume that these cell aggregates were derived from detached subretinal fibrosis or subretinal bands in the area of retinal detachment. However, most of the subretinal fibrosis appeared to be hyperfluorescent in the FAF images due to the separation of the outer retina from the retinal pigment epithelium. This then prevented the phagocytosis of the photoreceptor outer segments with increased accumulation of outer segments on the outer retina and subretinal space [22, 23]. Thus, these cell aggregates are most likely derived from RPE cells that migrated into the subretinal space of the detached retina.

Our study has several limitations. First, the types of retinal detachments in the two groups varied. In addition, some images were not present in the medical records and could not be obtained because of the retrospective nature of this study. Second, the number of patients, especially the eyes with IR hyperreflectivity, was too few to perform meaningful statistical analyses. Third, the follow-up period of 6 months may be too short to make a conclusion of the types of SRPs.

## Conclusions

The incidence of SRPs was higher in eyes with macular-off RRD, with subretinal precipitates, and with shorter axial lengths. The SRPs consisted of lipofuscin-related hyperfluorescent subretinal fluid that appeared as multiple subretinal fluid blebs. There are also melanin-related bright IR particles from the proliferation of RPE cells.

## Supplementary Information

**Additional file 1. **Intraoperative video during pars plana vitrectomy for retinal detachment (Videoclip-1: the same eye in Fig. 2).

## Data Availability

The data presented in this study are available on request from the corresponding author (MI).

## Abbreviations

PPV: Pars plana vitrectomy
RRD: Rhegmatogenous retinal detachment
RPE: Retinal pigment epithelium
OCT: Optical coherence tomography
IR: Infrared reflectance
FAF: Fundus autofluorescence
BCVA: Best-corrected visual acuity
logMAR: Logarithm of the minimum angle of resolution
TA: Triamcinolone acetonide
PVD: Posterior vitreous detachment
SF_6_: Sulfur hexafluoride
